# Identification of angiogenic properties of insulin-like growth factor II in in vitro angiogenesis models

**DOI:** 10.1054/bjoc.1999.0931

**Published:** 2000-01-18

**Authors:** O H Lee, S K Bae, M H Bae, Y M Lee, E J Moon, H J Cha, Y G Kwon, K W Kim

**Affiliations:** Department of Molecular Biology, Pusan National University, Pusan, 609-735, Korea; Institute of Environment & Life Science, Hallym University, Chunchon, 200-702, Korea

**Keywords:** insulin-like growth factor II, tube formation, migration, invasion, HUVECs

## Abstract

Insulin-like growth factor II (IGF-II), highly expressed in a number of human tumours, has been recently known to promote neovascularization in vivo. Yet, the detailed mechanism by which IGF-II induces angiogenesis has not been well defined. In the present study, we explored an angiogenic activity of IGF-II in in vitro angiogenesis model. Human umbilical vein endothelial cells (HUVECs) treated with IGF-II rapidly aligned and formed a capillary-like network on Matrigel. In chemotaxis assay, IGF-II remarkably increased migration of HUVECs. A rapid and transient activation of p38 mitogen-activated protein kinase (p38 MAPK) and p125 focal adhesion kinase (p125^FAK^) phosphorylation was detected in HUVECs exposed to IGF-II. IGF-II also stimulated invasion of HUVECs through a polycarbonate filter coated with Matrigel. Quantitative gelatin-based zymography identified that matrix metalloproteinase-2 (MMP-2) activity generated from HUVECs was increased by IGF-II. This induction of MMP-2 activity was correlated with Northern blot analysis, showing in HUVECs that IGF-II increased the expression of MMP-2 mRNA, while it did not affect that of TIMP-2, a tissue inhibitor of MMP-2. These results provide the evidence that IGF-II directly induces angiogenesis by stimulating migration and morphological differentiation of endothelial cells, and suggest that IGF-II may play a crucial role in the progression of tumorigenesis by promoting the deleterious neovascularization. © 2000 Cancer Research Campaign


*British Journal of Cancer* (2000) **82**(2), 385–391
© 2000 Cancer Research Campaign
Article no. bjoc.1999.0931

### Identification of angiogenic properties of insulin-like growth factor II in in vitro angiogenesis models

**OH Lee[1], SK Bae[1], MH Bae[1], YM Lee[1], EJ Moon[1], HJ Cha[1], YG Kwon[2] and KW Kim[1]**

[1]Department of Molecular Biology, Pusan National University, Pusan 609-735, Korea; [2]Institute of Environment & Life Science, Hallym University, Chunchon 200-702, Korea

**Summary** Insulin-like growth factor II (IGF-II), highly expressed in a number of human tumours, has been recently known to promote neovascularization in vivo. Yet, the detailed mechanism by which IGF-II induces angiogenesis has not been well defined. In the present study, we explored an angiogenic activity of IGF-II in in vitro angiogenesis model. Human umbilical vein endothelial cells (HUVECs) treated with IGF-II rapidly aligned and formed a capillary-like network on Matrigel. In chemotaxis assay, IGF-II remarkably increased migration of HUVECs. A rapid and transient activation of p38 mitogen-activated protein kinase (p38 MAPK) and p125 focal adhesion kinase ($p125^{FAK}$) phosphorylation was detected in HUVECs exposed to IGF-II. IGF-II also stimulated invasion of HUVECs through a polycarbonate filter coated with Matrigel. Quantitative gelatin-based zymography identified that matrix metalloproteinase-2 (MMP-2) activity generated from HUVECs was increased by IGF-II. This induction of MMP-2 activity was correlated with Northern blot analysis, showing in HUVECs that IGF-II increased the expression of MMP-2 mRNA, while it did not affect that of TIMP-2, a tissue inhibitor of MMP-2. These results provide the evidence that IGF-II directly induces angiogenesis by stimulating migration and morphological differentiation of endothelial cells, and suggest that IGF-II may play a crucial role in the progression of tumorigenesis by promoting the deleterious neovascularization. © 2000 Cancer Research Campaign

**Keywords:** insulin-like growth factor II; tube formation; migration; invasion; HUVECs

Angiogenesis, the development of new blood vessels from quiescent endothelium, is an inevitable process in a variety of physiological conditions including wound healing, embryonic development and organ regeneration. Conversely, persistent uncontrolled angiogenesis are known to contribute to the progression of many human diseases such as tumour, diabetic retinopathy and rheumatoid arthritis (Folkman, 1985). In particular, the expansion of solid tumours beyond 2–3 mm in size is critically dependent on the formation of new blood vessels of supply nutrients and oxygen (Hanahan and Folkman, 1996). The process of angiogenesis is complex and involves several discrete events, such as proliferation and migration of endothelial cells, and extracellular matrix (ECM) degradation. Up to date, a wide variety of growth factors and cytokines have been identified as positive or negative regulators of angiogenesis.

Insulin-like growth factor (IGF)-II, a polypeptide which has structural homology with insulin and IGF-I, is known to play a role in regulating proliferation and differentiation in a variety of cell types (Cohick and Clemmons, 1993). In fetal development, IGF-II was recognized as a crucial fetal growth factor, as was identified in high concentrations in multiple fetal tissues (Bowsher et al, 1991) and the disruption of IGF-II gene in mice resulted in substantial growth retardation (DeChiara et al, 1990). IGF-II also stimulated the differentiation of neuronal and muscle cells. The effects of IGF-II on cells were exerted by its interaction with three major cell surface receptors: the insulin receptor, the type-I IGF receptor (IGF-IR), and type-II IGF receptor (IGF-IIR) and the activity of IGF-II is also modulated by specific IGF binding proteins. IGF-II binds with high affinity to the IGF-IIR even though IGF-II binds with lower affinity to the insulin receptors and the IGF-IR. Two latter receptors are structurally similar and have an intrinsic tyrosine kinase activity. The mitogenic activity of IGF-II is generally considered to be mediated by an interaction with IGF-IR (Storchenfeldt et al, 1991). IGF-IIR is distinct from two other IGF receptors, and possesses a short cytosolic tail that lacks an intrinsic tyrosine kinase activity (Stewart and Rotwein, 1996). Concerning with the intracellular signalling pathways transferred by IGF-IIR is not clearly identified. However, several lines of evidence have been reported that IGF-IIR may be coupled to G protein (Nishimoto, 1993). Recently, Groskopf et al (1997) demonstrated in bovine capillary endothelial cells that proliferin-induced chemotaxis mediated by an interaction of this hormone with IGF-IIR occurs through a G protein coupled pathway, and that the chemotactic response requires the MAPK activation.

The involvement of IGF-II has been implicated in the growth regulation of human tumours (Daughaday, 1990). Many researchers have observed independently that IGF-II was highly expressed in a number of human tumours, which include Wilms' tumour (Reeve et al, 1985), breast tumour (Cullen et al, 1991), neuroblastoma (Sullivan et al, 1995) and rhabdomyosarcoma (Minniti et al, 1994). In addition, IGF-II is known to stimulate the growth of hepatocellular carcinoma (HCC) cells in vitro (Kraft et al, 1993). We also found previously that most cirrhotic and HCC tissues express IGF-II, and in a subsequent study we demonstrated that IGF-II secreted from HCC functions as an angiogenic factor directly as well as indirectly through increase in vascular endothe-

*Received 5 May 1999*
*Revised 8 September 1999*
*Accepted 9 September 1999*

*Correspondence to:* KW Kim lial growth factor (VEGF) production (Kim et al, 1998). The direct angiogenic activity of IGF-II in rat cornea was also observed by Volpert et al (1996). Therefore, the importance of IGF-II as an angiogenic factor has been emphasized. However, the specific molecular function of IGF-II in promoting angiogenesis remains to be further investigated.

In the present study, the mechanisms of IGF-II in the regulation of angiogenesis were studied in HUVECs. We demonstrate that IGF-II induces angiogenesis by directly stimulating endothelial cell migration and tube formation. In addition, IGF-II stimulates invasiveness of HUVECs into the basement membrane (BM) through up-regulation of the expression of proteolytic enzyme, MMP-2.

### MATERIALS AND METHODS

### Cell culture

HUVECs were obtained from the American Type Culture Collection. The cells were maintained in a gelatin-coated 75-cm$^2$ flask in M199 (Life Technologies) supplemented with 20% fetal bovine serum (FBS), 100 units ml$^{-1}$ penicillin, 100 µg ml$^{-1}$ streptomycin, 3 ng ml$^{-1}$ basic fibroblast growth factor (bFGF) (Life Technologies), and 5 units ml$^{-1}$ heparin (Life Technologies) at 37°C under 5% carbon dioxide, 95% air. IGF-II was purchased from R&D Systems Inc.

### [$^3$H]Thymidine incorporation assay

HUVECs were seeded at a density of $1 \times 10^4$ cells per well in 24-well plate. Cells were incubated in growth media and allowed to attach for 24 h. Cells were washed two times with M199 and incubated for 12 h in M199 containing 1% FBS. Cells were stimulated by the addition of the indicated concentration of IGF-II or 10 ng ml$^{-1}$ of bFGF for 4 days, followed by the addition of 1 µCi ml$^{-1}$ [$^3$H]thymidine for 5 h. High molecular mass [$^3$H]-radioactivity was precipitated using 5% trichloroacetic acid at 4°C for 30 min. After two washes with ice-cold water, [$^3$H]-radioactivity was solubilized in 0.2 N sodium hydroxide and 0.1% sodium dodecyl sulphate (SDS), and determined by liquid scintillation counter.

### Growth of HUVECs on Matrigel

A total of 250 µl of Matrigel (Collaborative Research) was pipetted into a 16 mm diameter tissue culture wells and polymerized for 30 min at 37°C (Albini et al, 1995). HUVECs were harvested with trypsin, resuspended in M199 with 10% FBS, plated onto a layer of Matrigel at a density of $4 \times 10^5$ cells per well, and followed by the addition of bFGF (30 ng ml$^{-1}$) and heparin (30 µg ml$^{-1}$) or IGF-II (100 ng ml$^{-1}$). Matrigel cultures were incubated at 37°C, After 24 h, the cultures were photographed.

### Chemotaxis assay

The chemotatic motility of HUVECs was determined by a modified Boyden chamber assay. Briefly, the lower surface of the polycarbonate filter (12 µm pore, Millipore) was coated with 10 µl of 0.5 mg ml$^{-1}$ type I collagen. IGF-II or bFGF, prepared in 600 µl of M199 with 1% FBS, was placed in the lower wells. HUVECs were trypsinized and suspended at a final concentration of $5 \times 10^5$ cells ml$^{-1}$ in M199 containing 1% FBS. Then, 400 µl of cell suspension was loaded into each of the upper wells. The chamber was incubated at 37°C for 4 h. Cells were fixed and stained with haematoxylin and eosin. Non-migrating cells on the upper surface of the filter were removed by wiping with a cotton swab, and chemotactic motility was quantified by counting the cells that migrated to the lower side of the filter with optical microscopy at ×400 magnification. Thirteen fields were counted for each assay. Each sample was assayed in triplicate, and the assays were repeated twice.

### In vitro invasion assay

In vitro invasion assay was performed as described for chemotaxis assay with exceptions that the upper side of filter was coated with 50 µl of 0.2 mg ml$^{-1}$ Matrigel and incubation time was 7 h.

### Immunoprecipitations

Confluent HUVECs were incubated for 24 h in M199 containing 1% FBS. The cells were treated with IGF-II or VEGF (Upstate Biotechnology) for 10 min, lysed at 4°C in 1 ml of a lysis buffer containing 10 mM Tris–HCl, pH 7.6, 5 mM EDTA, 50 mM sodium chloride (NaCl), 50 mM β-giycerophosphate, 50 mM sodium fluoride, 0.1 mM $Na_3Vo_4$, 1 mM phenylmethylsulphonyl fluoride, 0.5% Nonidet P-40, and 1% Triton X-100. Lysates were clarified by centrifugation at 15 000 ***g*** for 10 min and precleared by incubation with protein A-agarose beads (Upstate Biotechnology) for 1 h at 4°C. After removal of protein A-agarose by brief centrifugation, the supernatants were transferred to fresh tubes for immunoprecipitation. Immunoprecipitation was performed by incubating lysates with 1 µg ml$^{-1}$ of anti-p125$^{FAK}$ antibody (Upstate Biotechnology) for 3 h at 4°C. Immunocomplexes were collected by incubating lysates with protein A-agarose beads for a further 1 h. Immunoprecipitates were washed three times with lysis buffer and further analysed by Western blotting.

### Western blotting

Cell lysates or immunoprecipitates from HUVECs were loaded into a 10% SDS-polyacrylamide gel electrophoresis (SDS-PAGE) gel, and transferred to polyvinyldifluoride membrane (Immobilon P, Millipore Corp.). The blocked membranes were then incubated with anti-phospho-specific p38 MAPK (New England Biolabs), anti-p38 MAPK (Santa Cruz Biotechnology), anti-phosphotyrosine (PY20, Transduction Laboratories), or anti-p125$^{FAK}$ antibody (Upstate Biotechology), and the immunoreactive bands were visualized using chemiluminescent reagent as recommended by Amersham Pharmacia Biotechnolgy. The signals of the bands were quantitated using a densitometer.

### Gelatin zymography

HUVECs were seeded at a density of $2 \times 10^6$ cells per well of a 6-well plate. After 2 days, cells were rinsed with serum-free medium twice, and then incubated in 3 ml of serum-free medium with or without IGF-II for 12 h. The amount of secreted proteins in the conditioned media was quantified by Bio-Rad protein assay (Bio-Rad). The conditioned media containing 3 µg of secreted proteins were analysed by gelatin-based zymography, using a slightly modified procedure from that of Herron et al (1986). The

© 2000 Cancer Research Campaign

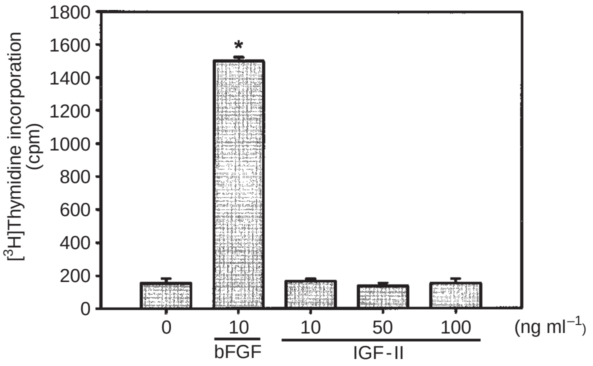

**Figure 1** The effect of IGF-II on DNA synthesis of HUVECs. IGF-II (10, 50, or 100 ng ml$^{-1}$) was added to HUVECs and incubated for 4 days. [$^3$H]Thymidine was present during the last 5 h of incubation. bFGF (10 ng ml$^{-1}$) was used as a positive control. The data represent the means ± s.e.m. of triplicate, and similar results were obtained in at least two different experiments. $P < 0.001$ from control

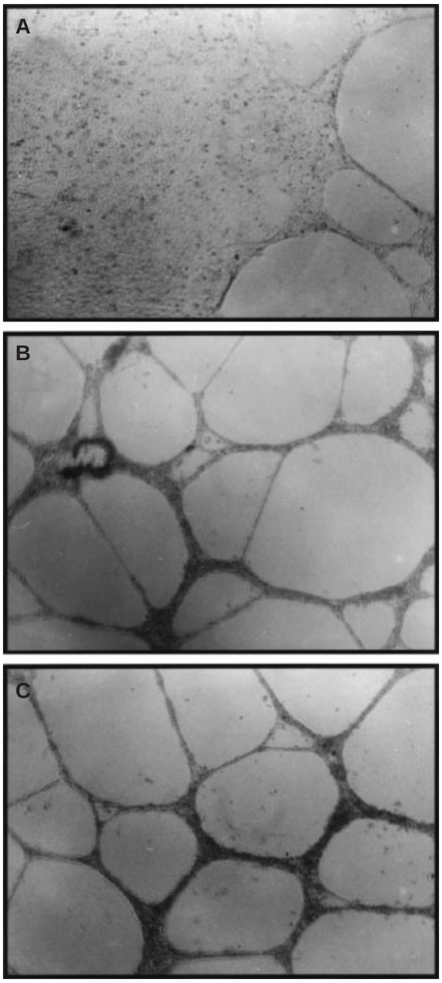

**Figure 2** IGF-II induces tube formation of HUVECs on Matrigel. HUVECs were plated on Matrigel-coated wells as described in Materials and Methods. Cultures were photographed after 24 h of incubation in the absence of activator (**A**), in the presence of 30 ng ml$^{-1}$ of bFGF and 30 µg ml$^{-1}$ of heparin (**B**), or in the presence of 100 ng ml$^{-1}$ of IGF-II (C) (×40)

proteins were separated by SDS-PAGE using 10% acrylamide co-polymerized with gelatin (0.33 mg ml$^{-1}$). After electrophoresis, the gel was rinsed twice with 2.5% Triton X-100 for 15 min and incubated for 18 h at 37°C in incubation buffer (0.05 M Tris–HCl (pH 7.5), 0.15 M NaCl, 0.01 M calcium chloride, 1 µM zinc chloride). Gelatinase was identified following staining of the gel in 0.25% Coomassie blue R250 and destaining in 7% acetic acid. The digested area appeared clear on a blue background, indicating the location of gelatinase.

### Northern blot analysis

Total RNAs were prepared from HUVECs using the Tri Reagent kit (Molecular Research Center). RNAs (30 µg) were electrophoresed on formaldehyde agarose gels, transferred to Zeta-Probe membranes (Bio-Rad), and probed with $^{32}$P-labelled MMP-2, TIMP-2, or β-actin cDNA fragments. The filter was prehybridized at 42°C for 1 h with hybridization buffer containing 50% deionized formamide, 7% SDS, 0.12 M $NaHPO_4$ and 0.25 M NaCl and then hybridized at 42°C overnight in hybridization buffer with denatured labelled probes. After hybridization, the filter was washed at 42°C in 2 × SSC (1 × SSC = 0.15 M NaCl and 0.015 M sodium citrate), 0.1% SDS for 15 min and 0.5 × SSC, 0.1% SDS for 15 min. The filter was then exposed to X-ray film. The signals of the bands were quantitated using a densitometer.

### Statistical analysis

Results are expressed as mean ± s.e.m. or as percentage ± s.e.m. of control. Statistical comparisons between groups were performed using the Student's *t*-test.

### RESULTS

### IGF-II induces vascular tube formation on Matrigel

Previously, we have demonstrated that IGF-II has a direct angiogenic activity in chick chorioallantoic membrane (CAM) assay (Kim et al, 1998). However, the detailed mechanisms involved in the regulation of angiogenesis by IGF-II have not been clearly identified. To analyse the direct angiogenic activities of IGF-II on endothelial cells, we have first examined the effect of IGF-II on proliferation of HUVECs by [$^3$H]thymidine incorporation assay. IGF-II did not affect the proliferation of HUVECs at the concentration of ranges from 10 ng ml$^{-1}$ to 100 ng ml$^{-1}$ while HUVEC proliferation was strongly stimulated by 10 ng ml$^{-1}$ bFGF (Figure 1). Higher concentration of IGF-II up to 1 µg ml$^{-1}$ had no effect on the proliferation of HUVECs (data not shown). Next, we examined the effect of IGF-II on the morphological differentiation of HUVECs on Matrigel. HUVECs resuspended in M199 containing 10% FBS without any other supplements were seeded at high density (4 × 10$^5$ cells) on plates coated with Matrigel. In this condition, HUVECs did not form complete network within 24 h (Figure 2A) and as usual it takes 3 days to form an organized tube-like structure. However, HUVECs stimulated with IGF-II (100 ng ml$^{-1}$) rapidly aligned and formed an anastomosing capillary-like network, and complete networks were observed after 24 h (Figure 2C). Thus, the effect of IGF-II on the morphology of HUVECs on Matrigel was similar to that of bFGF (Figure 2B). The rapid formation of tube network by IGF-II suggests that IGF-II may increase the migratory and invasive activities of HUVECs.

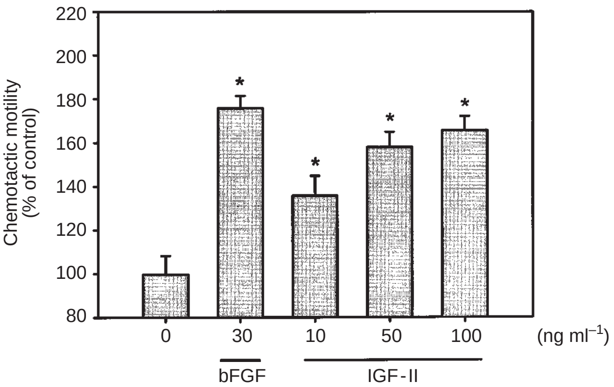

**Figure 3** IGF-II induces chemotactic migration of HUVECs. HUVECs were plated on polycarbonate filter of the upper compartment and exposed for 4 h to 30 ng ml$^{-1}$ bFGF or the indicated concentration of IGF-II added to the lower compartment. The detailed experimental procedures are described in Materials and Methods. Results are expressed as percentage ± s.e.m. of control. Each sample was assayed in triplicate, and the assays were repeated twice. $P < 0.001$ from control

### IGF-II induces HUVEC migration

To further investigate the mechanism associated with IGF-II-stimulated tube formation, we examined the effect of IGF-II on HUVEC migration using a modified Boyden chamber assay. As shown in Figure 3, IGF-II stimulated the chemotactic motility of HUVECs in a dose-dependent manner and the migratory activity at 100 ng ml$^{-1}$ of IGF-II was 60% increase over the control. However, the stimulatory effect of IGF-II on chemotactic motility of HUVECs was slightly weaker than that of bFGF (30 ng ml$^{-1}$).

### IGF-II induces p38 MAPK activation and p125$^{FAK}$ phosphorylation

Recently, it has been reported that the p38 MAPK activity mediates actin reorganization and cell migration whereas activation of extracellular signal-regulated protein kinases (ERKs) is associated with cell proliferation in HUVECs (Rousseau et al, 1997). Thus, we postulated the possibility that IGF-II may stimulate endothelial cell migration through p38 MAPK activation. To assess the effect of IGF-II on p38 MAPK, we exposed confluent HUVECs to various concentrations of IGF-II and measured the activation of p38 MAPK with rabbit polyclonal phospho-specific anti-p38 MAPK, which only detects phosphorylated and activated form. The specificity of phospho-specific anti-p38 MAPK was determined by bFGF (10 ng ml$^{-1}$, for 10 min) and VEGF (5 or 10 ng ml$^{-1}$, for 10 min) which are known stimuli of p38 MAPK in HUVECs. As shown in Figure 4A, cells treated with either bFGF or VEGF significantly increased the signals from immunoblot, indicating that this antibody is specific to phosphorylated form of p38 MAPK. The activation of p38 MAPK by IGF-II was evident at 10 ng ml$^{-1}$ and gradually increased in a dose-dependent manner (Figure 4B). In contrast, IGF-II even at 300 ng ml$^{-1}$ did not affect

A

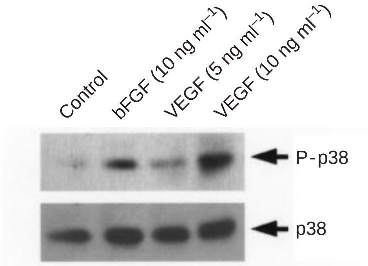

B

C

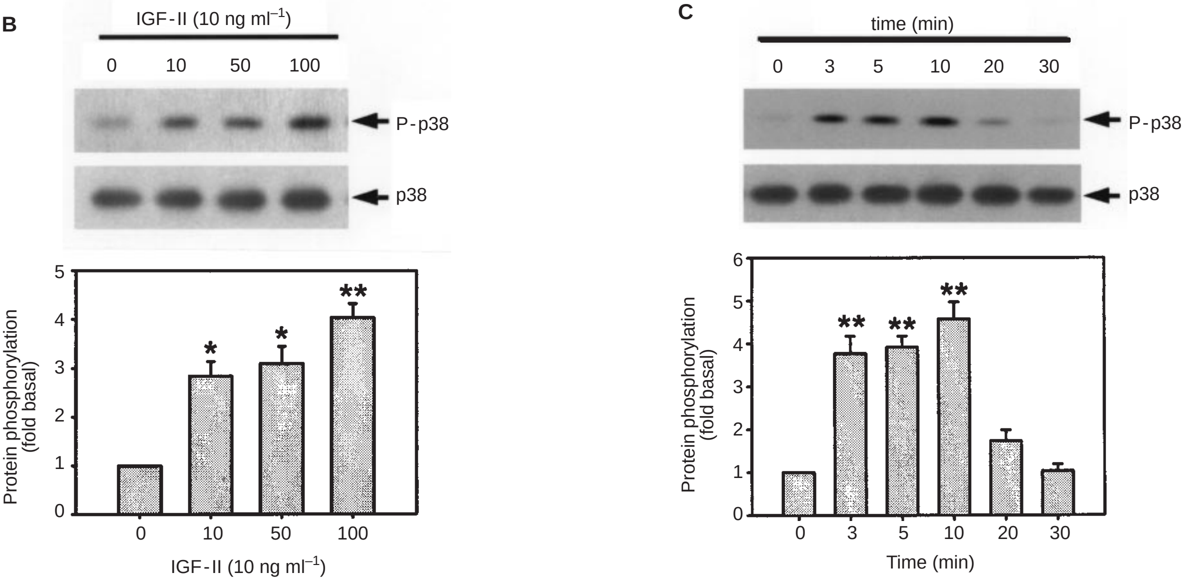

**Figure 4** IGF-II induces activation of p38 MAPK in HUVECs. (**A**) Effect of bFGF or VEGF on activation of p38 MAPK. Confluent HUVECs were stimulated for 10 min with or without bFGF (10 ng ml$^{-1}$) and VEGF (5 or 10 ng ml$^{-1}$). (**B**) Dose-dependent p38 MAPK activation. HUVECs were stimulated with various concentrations of IGF-II for 10 min. (**C**) Time courses of IGF-II-induced p38 MAPK activation. HUVECs were stimulated with 100 ng ml$^{-1}$ of IGF-II for the indicated times. p38 MAPK activation was determined by Western blot analysis using anti-phospho-specific p38 MAPK antibody (P-p38), which only detects phosphorylated and activated from of p38 MAPK. The amount of proteins was checked by reprobing the membrane with antibody to p38 MAPK (p38). The graphs represented quantitative results from densitometeric analysis. Values shown are the mean ± s.e.m. of three independent experiments and are expressed as fold-stimulation above control. *$P < 0.005$; ** $P < 0.001$ from control

© 2000 Cancer Research Campaign

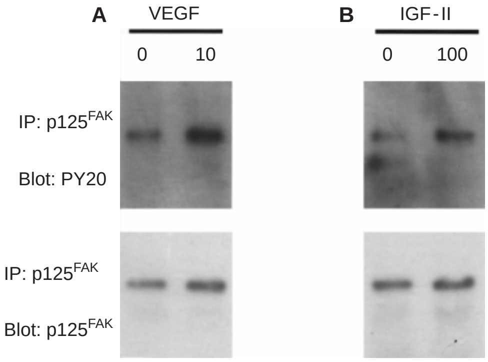

**Figure 5** IGF-II stimulates p125$^{FAK}$ tyrosine phosphorylation in HUVECs. Confluent cultures of HUVECs were treated with 10 ng ml$^{-1}$ VEGF (**A**) or 100 ng ml$^{-1}$ IGF-II (**B**) for 10 min. All cells used were lysed, and anti-p125$^{FAK}$ immunoprecipitates (IP) were prepared and immunoblotted with anti-phosphotyrosine antibody PY20 or anti-p125$^{FAK}$ antibody

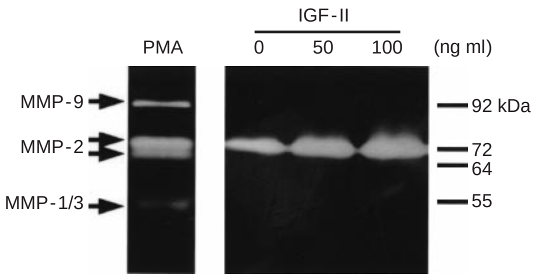

**Figure 7** Gelatin zymography of the culture medium of HUVECs treated with IGF-II. After treatment with 50 or 100 ng ml$^{-1}$ of IGF-II for 12 h, the culture media were used in gelatin-based electrophoresis and stained with Coomassie brilliant blue. The culture medium from HUVECs treated with PMA (40 ng ml$^{-1}$ for 12 h) was used for distinguishing the types of MMPs (lane 1)

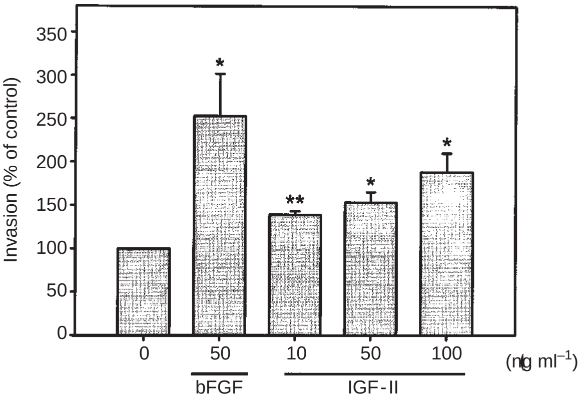

**Figure 6** IGF-II stimulates invasion of HUVECs. HUVECs were treated with the indicated concentrations of IGF-II. The cells invaded into BM and migrated to the lower side of the filter were counted. A total of 50 ng ml$^{-1}$ bFGF was used as a positive control. Results are expressed as percentages of control ± s.e.m. *$P < 0.05$; ** $P < 0.001$ from control phosphorylation of ERK1/2 that was, however, strongly activated by bFGF and VEGF (data not shown). This is well consistent with our observation that IGF-II has no effect on HUVEC proliferation. The activity of p38 MAPK was determined in HUVECs exposed to 100 ng ml$^{-1}$ of IGF-II at various periods of time. IGF-II induced a rapid activation of p38 MAPK that reached a peak of fourfold over basal level and maintained until 10 min (Figure 4C).

We have next investigated whether IGF-II activated p125$^{FAK}$ whose activation is correlated with cell adhesion and migration. Confluent HUVECs were treated with 100 ng ml$^{-1}$ of IGF-II for 10 min and anti-p125$^{FAK}$ immunoprecipitates were prepared and blotted by anti-phosphotyrosine antibody. IGF-II stimulated tyrosine phosphorylation of p125$^{FAK}$ by twofold over the control (Figure 5). The effect of IGF-II was similar to that of VEGF (10 ng ml$^{-1}$).

### IGF-II induces HUVECs to degrade and traverse the BM through the regulation of expression of MMP-2

To form new blood vessels, the migrating endothelial cells must break and traverse their own BM (Sage, 1997). To study the ability of IGF-II to stimulate invasion, we coated the polycarbonate filter with Matrigel preventing the migration of noninvasive cells (Albini et al, 1987). HUVECs were seeded on the filter and allowed to invade: bFGF (50 ng ml$^{-1}$) stimulated strongly the invasion of HUVECs. As similar to bFGF, IGF-II significantly promoted the invasion of HUVECs at the concentration of ranges from 10 ng ml$^{-1}$ to 100 ng ml$^{-1}$ (Figure 6). An essential pattern of this invasion includes degradation of the BM. MMPs are a family of inducible enzymes that degrade ECM components, allowing cells to traverse BM efficiently. Therefore, we performed gelatin zymography to examine the effect of IGF-II on the expression of MMP-1, 2, 3 and 9. Analysis of serum-free conditioned medium of non-stimulated HUVECs showed the presence of gelatinolytic activity at 72 kDa MMP-2. IGF-II increased the expression of MMP-2 by 2.5-fold at a concentration of 100 ng ml$^{-1}$, but it had no effect on the expression of MMP-1, 3 and 9 (Figure 7). We used the conditioned medium of phorbol 12-myristate 13-acetate (PMA)-stimulated HUVECs for distinguishing the type of MMPs (Hanemaaijer et al, 1993). PMA at a concentration of 40 ng ml$^{-1}$ induced several MMPs including MMP-9 (92 kDa), active form of MMP-2 (64 kDa) and MMP-1/MMP-3 (55 kDa).

To confirm the induction of MMP-2 by IGF-II, we conducted Northern blot analysis using specific cDNA probes for MMP-2 and its inhibitor, TIMP-2. IGF-II (100 ng ml$^{-1}$ for 12 h) induced a threefold increase in the expression of MMP-2 mRNA (Figure 8A). However, IGF-II had no significant effect on the expression of TIMP-2 mRNA in HUVECs (Figure 8B). These results suggest that the invasion activity of HUVECs by IGF-II may involve the changed ratio of the levels of MMP-2 and TIMP-2.

### DISCUSSION

High levels of IGF-II are found in a number of human tumours including human HCC (Kim et al, 1998), Wilms' tumour (Reeve et al, 1985), breast tumour (Cullen et al, 1991), neuroblastoma (Sullivan et al, 1995) and rhabdomyosarcoma (Minniti et al, 1994). In these tumours, IGF-II is known to contribute to the tumour growth. Previously, we reported that IGF-II, expressed in nearly all of the cases of cirrhotic and HCC tissues, plays an important role in the development of neovascularization of HCC. IGF-II indirectly induced angiogenesis during HCC by increasing the expression of VEGF in HCC cells and directly stimulated angiogenesis in the CAM (Kim et al, 1998). In the present study,

*© 2000 Cancer Research Campaign*

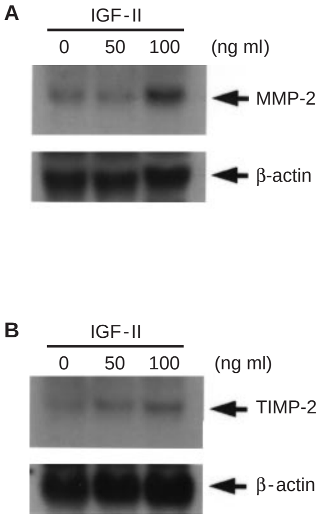

**Figure 8** Expression of MMP-2 and TIMP-2 in HUVECs treated with IGF-II. A total of 30 μg of total RNA isolated from HUVECs treated with 50 or 100 ng $ml^{-1}$ of IGF-II for 12 h were analysed by Northern blot. The blots were hybridized with $^{32}P$-labelled cDNA probes of MMP-2 (**A**) and TIMP-2 (**B**). To assess loading differences, radiolabelled DNA was stripped from blot and filters were reprobed with β-actin probe we have further demonstrated that IGF-II promotes the morphological differentiation of HUVECs into capillary-like structures on Matrigel. This phenomenon is supported by the findings that IGF-II remarkably increases cell migration and invasiveness. Moreover, the mechanisms of these processes have been further elucidated that IGF-II rapidly induced the activation of p38 MAPK and $p125^{FAK}$ phosphorylation, which may be involved in the signalling pathways of regulating endothelial cell migration, and substantially increased the expression of MMP-2, a major ECM degrading enzyme expressed in HUVECs.

The overall mechanism of completing angiogenesis *in vivo* requires an integral activation of endothelial cells, which includes proliferation, migration, matrix invasion and tube formation (Sage, 1997). IGF-II seemed not to be an endothelial cell mitogen since it did not significantly stimulate DNA synthesis in at least two types of endothelial cells, HUVECs in this study (Figure 1) and bovine aortic endothelial cells by others (Bar et al, 1988).

In contrast to the lack of IGF-II activity in endothelial cell proliferation, IGF-II strongly stimulated the capillary-like tube formation of HUVECs on plates coated with Matrigel, which is a reconstituted basement membrane (Figure 2). Endothelial cells cultured on Matrigel can slowly form tubular network without any other supplements because Matrigel has minimal amount of angiogenic components such as laminin and bFGF. When high density (~80%) of HUVECs were seeded on Matrigel, incomplete network was formed within 24 h without any other supplements. However, HUVECs treated with IGF-II rapidly moved, aligned and formed a complete network within 24 h, with similar kinetics to that of 10 ng $ml^{-1}$ bFGF. Thus, it is most likely that the direct angiogenic activity of IGF-II previously observed in the quantitative CAM assay (Kim et al, 1998) may be conferred by the stimulatory activity on endothelial cell migration and differentiation rather than proliferation.

Indeed, we observed that IGF-II markedly stimulated the migration of HUVECs as might have been expected (Figure 3). Similarly, Volpert et al (1996) demonstrated that the placental angiogenic hormone proliferin stimulates bovine capillary endothelial cells (BCECs) migration in vitro and neovascularization in the rat cornea by an interaction of this hormone with cell surface IGF-IIR and IGF-II also induces BCEC migration through its interaction with IGF-IIR rather than IGF-IR. In a subsequent study, Groskopf et al (1997) suggested that chemotaxis initiated by either proliferin or IGF-II binding to IGF-IIR occurs through a G protein-coupled signalling pathway leading to the ERK activation. In this study, we show that IGF-II noticeably induces the biphasic activation of p38 MAPK with respect to time of treatment with IGF-II in HUVECs (Figure 4) and also 100 ng $ml^{-1}$ of IGF-II stimulated the tyrosine phosphorylation of $p125^{FAK}$ with similar potency to that by 10 ng $ml^{-1}$ of VEGF (Figure 5). The involvement of MAPK pathways in cell migration was recently identified (Rousseau et al, 1997). In HUVECs, the treatment of VEGF induced actin reorganization, the formation of focal adhesion, and cell migration, and those were inhibited by pretreatment with the specific inhibitor of p38 MAPK, SB203580, but inhibiting the VEGF-induced activation of ERK with PD098059 had no effect. Furthermore, it has been suggested that VEGF-induced $p125^{FAK}$ phosphorylation occurs through a pathway independent of ERK activation (Abedi and Zachary, 1997). It is noteworthy that there was a discrepancy in requirement of ERK pathway between VEGF-induced HUVECs migration and IGF-II-induced BCEC migration. This may be due to the differences in either cell types or stimuli. On the basis of our observations, it would be predicted the possibility that the molecular mechanism by which IGF-II stimulates the migration of HUVECs may partly involve the formation of focal adhesion mediated by p38 MAPK activation and $p125^{FAK}$ phosphorylation, similar to VEGF-induced focal adhesion formation. However, much more remained to be investigated for the roles of p38 MAPK and other signalling molecules leading to HUVEC migration by IGF-II.

IGF-II-induced tube formation ability of HUVECs involves the increase of invasiveness into the BM (Figure 6). The present data demonstrated that IGF-II treatment increases the ability of HUVECs to invade BM. An essential pattern of this process includes degradation of the BM. Many proteolytic enzymes secreted from endothelial cells during the progression of angiogenesis have been reported to degrade components of the ECM and BM. These proteolytic enzymes include MMP-1 (collagenase-1), MMP-2 (gelatinase A; 72 kDa type IV collagenase), MMP-3 (stromelysin 1) and MMP-9 (gelatinase B; 92 kDa type IV collagenase) and can degrade native collagens, gelatin and other ECM components. Interstitial collagens (type I, II and III), present in the ECM, are degraded in a two-step process involving an initial cleavage by MMP-1, followed by further degradation by MMP-2, MMP-3 or MMP-9 (Matrisian, 1992). MMP-1, 2, 3, and 9 are known to be expressed in HUVECs (Hanemaaijer et al, 1993). Quiescent HUVECs showed an intrinsic MMP-2 activity and weak activities of MMP-1/3. In gelatin-based zymography, a $M_r$ 72 000 band of gelatinolysis, which is assigned to MMP-2 activity, was noticeably increased in the conditioned media from HUVECs treated with IGF-II compared to the control media (Figure 7). This result suggested that IGF-II might up-regulate the expression of MMP-2 protein. To confirm the increase in MMP-2 activity by IGF-II, we have investigated the mRNA expression of MMP-2 and TIMP-2, a tissue inhibitor of MMP-2. TIMP-2 is known to form 1:1 complexes with MMP-2 and that regulates the enzymatic activity of MMP-2. Northern blot analysis (Figure 8) revealed that treatment of HUVECs with IGF-II markedly up-regulated the

 *© 2000 Cancer Research Campaign*

mRNA expression of MMP-2, but not significantly TIMP-2. These results are inconsistent with gelatin zymography analysis and indicated that the increase in MMP-2 activity by IGF-II was due to the increased expression of MMP-2. Thus, it is suggested that the stimulatory effect of IGF-II on HUVEC invasion into BM can be partly attributable to a breakdown of net proteolytic balance, at least involving up-regulation of MMP-2 activity. The signalling pathways through which IGF-II elicits the expression of MMP-2 gene remain to be unidentified. Further experimental work is necessary to examine the correlation between activation of p38 MAPK and MMP-2 expression by IGF-II in HUVECs.

In summary, we have demonstrated that IGF-II directly induces angiogenesis by stimulating cell migration, invasion and tube formation. These results together with our previous observations confer further evidence that the high level of IGF-II expression in tumour may play a crucial role in the progression of tumorigenesis by promoting the deleterious neovascularization.

### ACKNOWLEDGEMENTS

We are grateful to Dr M Seiki for providing MMP-2 and TIMP-2 cDNA probes. This research was supported by the Hallym Academy of Sciences, Hallym University, and the National Research Laboratory fund, the Ministry of Science and Technology, Korea

### REFERENCES

Abedi H and Zachary I (1997) Vascular endothelial growth factor stimulates tyrosine phosphorylation and recruitment to new focal adhesions of focal adhesion kinase and paxillin in endothelial cells. *J Biol Chem* **272:** 15442–15451

Albini A, Barillari G, Benell R, Gallo RC and Ensoli B (1995) Angiogenic properties of human immunodeficiency virus type 1 Tat protein. *Proc Natl Acad Sci USA* **92**: 4838–4842

Albini A, Iwamoto Y, Kleinman HK, Martin GR, Aaronson SA, Kozlowski JM and McEwan RN (1987) A rapid in vitro assay for quantitating the invasive potential of tumour cells. *Cancer Res* **47**: 3239–3245

Bar RS, Siddle K, Dolash S, Boes M and Dake B (1988) Actions of insulin and insulin-like growth factors I and II in cultured microvessel endothelial cells from bovine adipose tissue. *Metabolism* **37**: 714–720

Bowsher RR, Lee WH, Apathy JM, O'Brien PJ, Ferguson AL and Henry DP (1991) Measurement of insulin-like growth factor-II in physiological fluids and tissues. I. An improved extraction procedure and radioimmunoassay for human and rat fluids. *Endocrinology* **128**: 805–814

Cohick WS and Clemmons DR (1993) The insulin-like growth factors. *Annu Rev Physiol* **55**: 131–153

Cullen KJ, Smith HS, Hill S, Rosen N and Lippman ME (1991) Growth factor messenger RNA expression by human breast fibroblasts from benign and malignant lesions. *Cancer Res* **51**: 4978–4985

Daughaday WH (1990) Editorial: the possible autocrine/paracrine and endocrine roles of insulin-like growth factors of human tumours. *Endocrinology* **127**: 1–4

Dechiara TM, Efstratiadis A and Robertson EJ (1990) A growth-deficiency phenotype in heterozygous mice carrying an insulin-like growth factor II gene disrupted by targeting. *Nature* **345**: 78–80

Folkman J (1985) Tumor angiogenesis. *Adv Cancer Res* **43**: 175–203

Groskopf JC, Syu L-J, Saltiel AR and Linzer DIH (1997) Proliferin induces endothelial cell chemotaxis through a G protein-coupled, mitogen-activated protein kinase-dependent pathway. *Endocrinology* **138**: 2835–2840

Hanahan D and Folkman J (1996) Patterns and emerging mechanisms of the angiogenic switch during tumorigenesis. *Cell* **86**: 353–364

Hanemaaijer R, Koolwijk P, Le Clercq L, De Vree WJ and Van Hinsbergh VWM (1993) Regulation of matrix metalloproteinase expression in human vein and microvascular endothelial cells. *Biochem J* **296**: 803–809

Herron GS, Banda MJ, Clark EJ, Gavrilovic J and Werb Z (1986) Secretion of metalloproteinases by stimulated capillary endothelial cells. II. Expression of collagenase and stromelysin activities is regulated by endogenous inhibitors. *J Biol Chem* **261**: 2814–2818

Kim K-W, Bae S-K, Lee O-H, Bae M-H, Lee M-J and Park BC (1998) Insulin-like growth factor II induced by hypoxia may contribute to angiogenesis of human hepatocellular carcinoma. *Cancer Res* **58**: 348–351

Kraft A, Reid LM and Zvibel I (1993) Suramin inhibits growth and yet promotes insulin-like growth factor II expression in HepG2 cells. *Cancer Res* **53**: 652–657

Matrisian LM (1992) The matrix-degrading metalloproteinases. *Bioessays* **14**: 455–463

Minniti CP, Tsokos M, Newton WA Jr and Helman LJ (1994) Specific expression of insulin-like growth factor-II in rhabdomyosarcoma tumor cells. *Am J Clin Pathol* **101**: 198–203

Nishimoto I (1993) The IGF-II receptor system: a G protein-linked mechanism. *Mol Reprod Dev* **35**: 398–406

Reeve AE, Eccles MR, Wilkins RJ, Bell GI and Millow LJ (1985) Expression of insulin-like growth factor-II transcripts in Wilms' tumour. *Nature* **317**: 258–260

Rousseau S, Houle F, Landry J and Huot J (1997) p38 MAP kinase activation by vascular endothelial growth factor mediate actin reorganization and cell migration in human endothelial cells. *Oncogene* **15**: 2169–2177

Sage EH (1997) Pieces of eight: bioactive fragments of extracellular proteins as regulators of angiogenesis. *Trends in Cell Biology* **7**: 182–186

Stewart CEH and Rotwein P (1996) Growth, differentiation, and survival: multiple physiological functions for insulin-like growth factors. *Physiol Rev* **76**: 1005–1026

Storchenfeldt L, Schofield PN and Engstrom W (1991) Stimulatory effect of insulin-like growth factor II on DNA synthesis in the human embryonic cornea. *Cell Biol Int Rep* **15**: 1217–1223

Sullivan KA, Castle VP, Hanash SM and Feldman EL (1995) Insulin-like growth factor II in the pathogenesis of human neuroblastoma. *Am J Pathol* **147**: 1790–1798

Volpert O, Jackson D, Bouck N and Linzer DI (1996) The insulin-like growth factor II/mannose 6-phosphate receptor is required for proliferin-induced angiogenesis. *Endocrinology* **137**: 3871–3876

© 2000 Cancer Research Campaign 
